# Role of Allogeneic Transplantation in Multiple Myeloma in the Era of New Drugs

**DOI:** 10.4084/MJHID.2010.013

**Published:** 2010-06-01

**Authors:** Benedetto Bruno, Luisa Giaccone, Moreno Festuccia, Mario Boccadoro

**Affiliations:** Division of Hematology at the S. Giovanni Battista Hospital, University of Torino, Via Genova 3, Torino, Italy

## Abstract

High-dose melphalan with autologous stem cell rescue has been regarded as the standard of care for patients with newly diagnosed myeloma up to the age of 65–70 years. The recent development of agents with potent anti-tumor activity such as thalidomide, lenalidomide and bortezomib has further improved overall survival and response rates. However, relapse is a continuous risk.

Allografting is a potentially curative treatment for a subset of multiple myeloma patients for its well documented *graft-vs-myeloma* effects. However, its role has been hotly debated. Even though molecular remissions have been reported up to 50% after high-dose myeloablative conditionings, their applications, given the high toxicity, have been for long limited to younger relapsed/refractory patients. These limitations have greatly been reduced through the introduction of non-myeloablative/reduced-intensity conditionings.

The introduction of new drugs, characterised by low risks of early mortality, indeed requires to define role and timing of an allograft to capture the subset of patients who may most benefit from *graft-vs-myeloma* effects.

Ultimately, new drugs should not be viewed as mutually exclusive with an allograft. They may be employed to achieve profound cytoreduction before and enhance *graft-versus-myeloma* effects as consolidation/maintenance therapy after an allograft. However, this combination should be explored only in well-designed clinical trials.

## Introduction:

Multiple myeloma is a fatal plasma cell disorder, though recent advances in the understanding of its pathogenesis has identified peculiar mechanisms that have become targets of agents with potent anti-myeloma activity such as lenalidomide and bortezomib. High-dose chemotherapy and autologous transplantation with/without these newer agents has been regarded as standard treatment for newly diagnosed younger patients. Disease recurrence is, however, a continuous risk. Allografting appears the only potentially curative treatment on account of well-documented *graft-vs-myeloma* effects.

**Figure 1.**
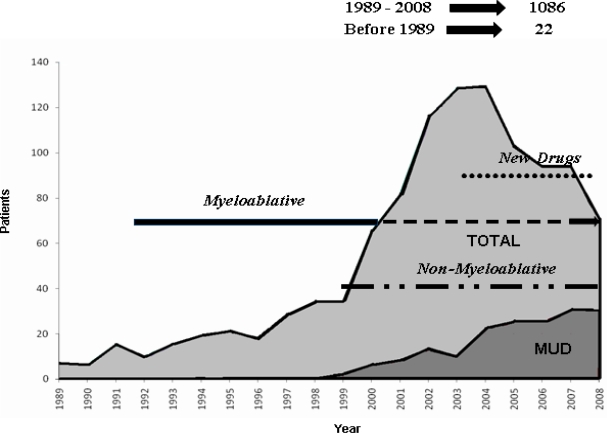
Number of transplants / year performed in Italy since 1989 through the Gruppo Italiano Trapianto di Midollo. The gray area represents transplants from unrelated donors (MUD). The clinical activity may be divided into three main periods: intense myeloablative conditionings were employed up to the late 90’; reduced-intensity and non-myeloablative conditionings greatly renewed the interest in allografting in the early 2000’. More recently, with the introduction of new drugs, the number of transplants has declined. However, the use of unrelated donors appears increased.

Between 1989 and 2008, 1089 allogeneic transplants were performed in Italy through the activity of the Gruppo Italiano Trapianto di Midollo (GITMO) (**Figure 1**). This clinical activity may be divided into three main periods. Up to the late 90’, intense myeloablative conditionings were employed. Given their high mortality and toxicity, their application was primarily limited to heavily pretreated patients at relapse or refractory to chemotherapies.

The introduction of reduced-intensity and non-myeloablative conditionings greatly renewed the interest in allografting and the incidence of this procedure peaked in the early 2000’. These regimens allowed to increase the eligible age for an allograft up to 70 years even in medically unfit patients. Moreover, the burden of myeloma eradication was shifted from chemotherapy to donor T cells.

More recently, with the introduction of new drugs, the number of transplants has declined even though the use of unrelated donors appears increased.

This manuscript aims at reviewing the current evidence of *graft-vs-myeloma* effects; the results obtained with conventional myeloablative and, more recently, with non-myeloablative conditionings; and the possible integration of so-called new drugs in the setting of allografting to improve clinical outcomes.

## Myeloablative conditioning regimens (Table 1):

The most commonly used myeloablative conditioning regimens included cyclophosphamide with total body irradiation or busulfan, or melphalan and total body irradiation.^1^–^13^

The high transplant-related mortality up to 60% limited this approach to young, medically fit patients.^1^–^3^ Causes of death comprised regimen-related, graft-vs-host disease (GvHD) and its transplant-related complications. Strong myeloma effects on baseline organ functions and severe immunodeficiency may be responsible for transplant-related mortality observed in other malignancies. Most representative experiences on the use of myeloablative conditioning regimens in multiple myeloma come from Seattle, the US Intergroup Trial S9321, and a European Bone Marrow Transplantation (EBMT) Registry study.^2^,^4^,^14^ The largest single-center experience comes from the Seattle group at the Fred Hutchinson Cancer Research Center.^2^,^5^ One-hundred-thirty-six heavily pre-treated or disease refractory patients received an allograft between 1987 and 1999 from related (84%) or unrelated donors (16%). A day-100 transplant-related mortality of 48% was reported. The 5-year survival was 22% with disease-free survival of 14%. In 34% of patients who achieved complete remission, overall and disease-free survivals at 5 years were 48% and 37%. Subgroup analyses showed that early transplant-related mortality was approximately 20% for patients with chemo-sensitive disease who were transplanted within one year from diagnosis.

A North-American prospective trial compared autografting with myeloablative allografting.^4^ The US intergroup trial (S9321) of early vs late autografting included a third option that allowe patients with HLA-identical siblings, under the age of 55, to undergo an allograft after melphalan and total body irradiation. This arm of the study was prematurly closed after the first 36 patients were enrolled given an excessively high transplant-related mortality of 53%. After a follow up of 7 years, however, the overall survivals were identical at 39% for both autologous and allogeneic recipients, while the progression-free survivals were 15% for autologous recipients as compared to 22% for allogeneic recipients, respectively. However, while the risk of relapse and death continues in the cohorts treated with an autograft, the overall survival curve for the allogeneic cohort reached a plateau with follow up extending to 10 years.

A large retrospective registry analysis by the EBMT group showed a remarkable improvement in overall survival in the late 90’ due to a reduction in transplant-related mortality through improved supportive care and more careful patient selection.^14^ In this analysis, 690 patients, median age at transplant 44 years, who underwent a myeloablative allograft were divided into two cohorts: patients who received a bone marrow allograft between 1983–93 and those between 1994–98. In this latter cohort, some patients also received granulocyte-colony-stimulating factor (G-CSF) mobilized peripheral blood hematopoietic cells. Transplant-related mortality at 6 and 24 months was lower in the cohort transplanted between 1994–1998 than between 1983–1993, 21% versus 38% and 30% versus 46%. The reduced toxicity was associated with an increase in overall and progression-free survivals at 3 years from 35% to 55% and from 7 to 19 months for patients transplanted between 1994–1998. Furthermore, no differences in clinical outcomes were observed between patients who received marrow and those who received peripheral blood hematopoietic cells.

The interpretation of these studies to draw definitive conclusions is extremely difficult as the reported patients were not included in prospective control trials. Most patients were heavily pretreated, were chemo-resistant at the time of transplant and received a variety of conditionings and GvHD prophylaxes. The most consistent finding, however, was the high treatment-related mortality.

Despite selection bias, it was widely assumed that better clinical outcomes were associated with patients with chemo-sensitive myeloma at transplant. In most studies, only 10–25% of patients eventually became long-term disease-free survivors and were possibly cured.

## Reduced-intensity and non-myeloablative conditioning regimens (Table 2):

Though higher in multiple myeloma, the transplant-related morbidity and mortality associated with myeloablative conditioning regimens and allografting for the treatment of hematological malignancies have always been a matter of concern. These clinical observations prompted investigators, in the late 90’, to explore highly immunosuppressive, though less myelosuppressive and less intense, conditionings which could possibly establish stable donor engraftment while reducing transplant-related organ toxicities. Pioneering studies were carried out in Seattle where it was shown that donor engraftment could be obtained with the sole combination of low dose non-myeloablative total body irradiation (200 cGy) and fludarabine, followed by peripheral blood stem cells and potent immunosuppression with cyclosporine and mycophenolate mofetil.^15^ Shortly thereafter, the tandem approach of an autologous transplant followed, 2–4 months later, by a non-myeloablative allograft was also designed for patients with newly diagnosed multiple myeloma.^16^ In 52 patients treated with this tandem modality the complete remission rate was 48% while progression free survival and overall survival were 48% and 69% respectively. The same “tandem concept” was also developed by Kroger et al using melphalan, fludarabine and anti-thymocyte globulin with related and unrelated donors.^17^

The tandem approach of an autologous transplant followed by a low dose non-myeloablative total body irradiation has become the most widely used conditioning for myeloma patients. The rationale for this tandem “autologous-allogeneic” approach was to separate in time the high-dose cytoreduction with melphalan at standard 200 mg/m^2^ and the graft-vs-myeloma effect with the potential of drastically reducing treatment-related toxicity and mortality.

Two large series from Seattle and Italy have recently reported on more than 200 patients using the tandem auto/allo strategy. Long-term clinical outcomes of 102 patents treated with this approach, after a follow up of 6.3 years, were recently reported by Rotta et al.^18^ However, unlike the first report by the same group, patients were not uniformly in first line treatment. Overall, 42% of patients developed grade II–IV acute GvHD and 74% experienced chronic GvHD. Transplant-related mortality at 5 years was 18%, mostly due to GvHD and/or infections. Overall response rate was 94%, with 65% and 29% of patients achieving complete and partial remissions respectively. Median overall survival was not reached and progression-free survival was 3 years. Estimated 5 year overall and progression free survivals were 64% and 36%. Results were recently reported also by the Gruppo Italiano Trapianti di Midollo.^19^ One-hundred newly diagnosed patients younger than 65 years were registered in a prospective multi-center study. Major strength of the study was the rigid enrolment of untreated myeloma patients who underwent the same vincristin, adriamycin and dexamethasone (VAD)-based induction before the autologous cytoreductive transplant. Primary objectives were overall and event-free survivals from diagnosis. After a median follow up of 5 years, overall survival was not reached and event free survival was 37 months. Incidences of acute and chronic GvHD were 38% and 50%, respectively. Complete remission, achieved in 53% of patients, or very good partial remission prior to allografting were significantly associated with achievement of post-transplant remission and longer event-free survival. Interestingly, in both studies from Seattle and from the Italy graft-vs-myeloma effects were not associated with clinical GvHD.

In recent years, several reduce-intensity regimens have been designed including melphalan, 100–140 mg/m^2^, with or without fludarabine, and intermediate-dose busulfan.^20^–^27^ Moreover, anti-thymocyte globulin or alemtuzumab have been employed in some trials to reduce GvHD.^20^,^21^ In a review of the EBMT registry, 26 different conditioning regimens, with/without T cell depletion, in 229 patients were reported.^28^,^29^ Almost 80% of patients received peripheral blood stem cells. Acute grade II–IV GVHD developed in 31% extensive chronic GVHD in 25%. Transplant-related mortality was rather low at 22%, however, 3 year overall survival and progression free survival were disappointing at 41% and 21%. Best clinical outcome was observed in those patients who were transplanted in first remission and did not receive more than one autograft. The use of alemtuzumab to prevent GVHD had a negative impact on transplant-related mortality, progression free survival and overall survival. Achievement of complete remission and occurrence of chronic GvHD were associated with prolonged progression free survival. It is imperative to underline that fact the patients cohorts were highly heterogeneous and study designs greatly differ. No definite conclusions could be drawn.

More recently, studies comparing allografting after reduced-intensity conditionings and autografting have been published. The concept of Mendelian or genetic randomization has been applied to the assessment of outcomes in patients with hematological disorders who were treated with allografting or other therapies.^30^–^33^ This concept relies on the biological process through which offspring randomly inherit genetic traits half from each parent so that one in four siblings is expected to have a potential HLA-identical sibling donor. The comparison by the intention-to-treat principle between patients with HLA-identical siblings, who can be assigned to allografting, and those without such siblings, and who cannot receive an allograft, is used as a surrogate for an unbiased randomization.

The first such study was reported by the French group. The study compared two trials which included high risk myeloma patients carrying elevated serum β2-microglobulin and del(13).^34^ All patients underwent an autograft after melphalan at 200 mg/m^2^. Sixty-five patients with HLA-identical sibling donors then received an allograft after a conditioning with busulfan, fludarabine and high-dose anti-thymocyte globulin, 12.5 mg/kg. Outcomes were compared with 219 high risk patients who were treated with a second autograft after melphalan at 220 mg/m^2^. Transplant-related mortality and response rates were not different. After a median follow-up of 2 years, overall and event free survivals were 35% and 25%, and 41% and 30% for the double autologous and the autologous-allogeneic cohorts, respectively. The Authors concluded that patients with high risk features may not benefit from a reduced-intensity allograft. This study was criticized for the inclusion of high dose anti thymocyte globulin, 12.5 mg/kg, in the conditioning regimen. As a matter of fact, though the incidence of chronic GvHD was 7%, the high dose of anti-thymocyte globulin may have highly prevented potentially curative graft-vs-myeloma effects. This study was also updated.^35^ By intent-to-treat analysis on all 284 patients, after a median follow-up of 56 months, event-free survival did not significantly differ between tandem autologous and a single autograft followed by a reduced–intensity allograft (median 22 versus 19 months, p 0.58). There was a trend for a superior overall survival in the tandem autologous cohort (median 48 versus 34 months, p 0.07).

Another study by Bruno et al. reported on 245 consecutive newly diagnosed myeloma patients, up to the age of 65 years, diagnosed between 1998–2004 where 162 out of 199 with at least one sibling were HLA-typed with their potential sibling donors.^36^ The novelty of the study was the treatment assignment in function of the presence/absence of an HLA-identical sibling donor. Patients received induction with VAD-based regimens followed by a standard autograft with melphalan. Eighty patients with at least one HLA-identical sibling were offered total body irradiation -based non-myeloablative conditioning followed by an allograft with G-CSF mobilized peripheral blood stem cells. Eighty-two patients without an HLA-identical sibling were assigned to receive a second autograft after high-dose, 140–200 mg/m^2^, or intermediate-dose, 100 mg/m^2^, of melphalan. After a median follow up of 45 months, overall and event-free survivals were significantly longer in patients with donors: 80 versus 54 months and 35 versus 29 months. By multivariate analysis, having an HLA-identical sibling was an independent variable significantly associated with longer overall and event-free survivals. Overall, 58 and 46 patients completed the tandem autologous-allogeneic and the tandem autologous programs, with complete remission rates of 55% versus 26%. Transplant-related mortality was 10% and 2% respectively. Median overall survival was not reached in the tandem autologous-allogeneic cohort and was 58 months in the tandem autologous cohort. Event-free survival was 43 and 33 months, respectively. Criticisms to the study were that only 58 and 46 patients in in the tandem autologous-allogeneic cohort and in the tandem autologous cohort, respectively, completed their assigned treatments and the relatively poor outcome of the patients assigned to the tandem autograft. This study was also updated after a median follow up of 6 years. Overall survival was not reached for the 80 patients with an HLA-identical sibling and was 52 months for those without, p=0.004; event free survival remained significantly longer in patients with HLA-identical siblings: 35 versus 29 months, p=0.009. Median overall survival was not reached in the 58 patients who completed the tandem autologous-allogeneic program and was 64 months in the 46 who completed the double autologous program, p=0.04. Event-free survival was 37 and 33 months p=0.06.

A third biologically randomized study was reported by the Spanish PETHEMA group.^37^ One-hundred-ten patients, after failing to reach at least near-complete remission after a first autograft, received either a second autograft (No. 85) or an allograft (No.25) after a reduced-intensity conditioning with melphalan and fludarabine. There was a higher complete remission rate, 40% versus 11%, p=0.001, and a trend towards a longer progression-free survival, median 31 months versus not reached, p=0.08, in the reduced-intensity group. Patients who underwent an allograft showed a trend towards a higher transplant-related mortality, 16% versus 5%, p=0.07, and no difference in overall and event-free survivals.

Finally, 4 large prospective randomized studies, the Blood and Marrow Transplant Clinical Trials Network (BMT-CTN) 0102 trial in the U.S.A.; the Dutch-Belgian Hemato-Oncology Cooperative Group (HOVON) trial,^38^ the EBMT trial^39^ and the study by the German DSMM group^40^ in Europe, have recently been presented.

The large BMT-CTN 0102 trial comparing double autologous transplant versus tandem autologous/non-myeloablative allogeneic transplant completed the accrual in March 2007. More than 150 patients were biologically randomized to the latter cohort. The results from this study are eagerly awaited and should be released in 2010.

In the HOVON 54 study, newly diagnosed patients with an HLA-identical sibling donor included in the HOVON 50 study, a phase 3 study for the evaluation of thalidomide combined with high-dose melphalan, were allowed to proceed to a non-myeloablative allogeneic transplant from 2 and 6 months after a standard autograft, whereas patients without a suitable donor were randomized to thalidomide or interferon maintenance. By intent-to-treat analysis, no difference in progression free survival and overall survival were observed with an interim analysis that included 126 patients with a donor and 141 patients without.^38^

In the EBMT trial, progression free survival at 60 months was 35% for the tandem auto/allo cohort as compared to 18% for double auto, and overall survival 65% and 57% respectively.^39^ This trend was observed in both deletion 13 and non-deletion 13 patients. Final analyses of the Hovon and of the EBMT trials are expected in 2010.

Another prospective study comparing double autologous transplant versus tandem autologous/reduced-intensity allogeneic transplant, after a conditioning with fludarabine and melphalan, has been reported by the German DSMM.^40^ This study only included patients with deletion 13q14. Transplants from HLA-matched unrelated donors were allowed. Preliminary data showed a higher complete remission rate in patients with deletion 13q14 who received an allograft as compared to the autologous group (59% versus 32 %.p. 0.003). However, the projected overall survival at 3 years was 70% for the double autologous group and 60% for the allogeneic group (P=0.22). In the latter, transplant-related mortality at 2 years was only 12.7% even though 60% of patients received an allograft from an unrelated donor.

## The potentially curative role of allografting: graft-vs-myeloma:

The potentially unique, curative role of allografting consist of the immune reaction of donor T cells against myeloma cells through the recognition of possibly disease-specific antigens. Evidence for the existence of such reactions was initially documented by the achievement of complete remissions after the discontinuation of immunosuppression or after the infusion of donor T lymphocytes in patients with recurrent disease post-transplant.^41^–^43^ Some Authors, however, reported that the strongest predictors for response to donor lymphocyte infusions were acute and chronic GvHD^44^–^47^ indicating that GvHD and graft-vs.-myeloma may share the same antigenic targets. Chronic GVHD has been associated with longer response duration and prolonged overall survival. Recently, the Gruppo Italiano Trapianti di Midollo (GITMO), however, reported that the development of chronic GVHD did not correlate with the remission rates and response duration.^19^ Thus, subclinical graft-vs.-host reactions, especially after a non-mieloablative conditioning, may occur in the absence of detrimental GVHD. Finally, further evidence for graft-vs-myeloma are the molecular remissions, prelude to possible complete eradication, that have been reported up to 50% of patients following allografting.^48^

## Role of “new drugs”:

So called “new drugs” have greatly changed the treatment options for multiple myeloma. Not only do they target malignant plasma cells but also affect their cross-talk with the marrow microenvironment due to several immunomodulatory properties. Interestingly, they modulate T cell subpopulations that may play a pivotal role in graft-vs-myeloma effects. Thus, their role in combination with allografting should be extensively investigated.

Thalidomide, lenalidomide and bortezomib have recently been included in a number of randomized clinical trials in both young and elderly patients.^49^–^52^ Response rates have significantly been improved even though longer follow-up is needed to evaluate the impact on long-term overall survival.

In the setting of allografting, these new drugs have first been employed in patients relapsing after allografting. The addition of thalidomide to donor lymphocyte infusions improved efficacy of salvage treatment without increasing GvHD.^53^

Remarkable results have been obtained with lenalidomide in a cohort of patients with progressive disease after reduced-intensity allografting.^54^–^55^ In a study, 14/15 (93%) patients responded; however, a severe flare of GVHD in some patients was observed. Lenalidomide has also been employed as maintenance treatment to enhance graft-vs-myeloa in a prospective phase II study by the HOVON group.^56^ After, an autologous/non-myeloablative tandem transplant, patients were given lenalidomide at the dose of 10 mg/day for 21 days and then 7 days of rest. Treatment was started between 1 and 6 months post-transplant in patients with no GvHD. Preliminary results showed a drop out rate of 41% primarily due to acute flare of severe GVHD that strongly correlated with the start of maintenance. Given this recently reported toxicity profile, the GITMO group is conducting a study where lenalidomide, employed as maintenance, is started at 6 months post non-myeloablative transplant in patients without signs and/or symptoms of chronic GVHD.

Bortezomib has also been shown to be effective in patients with relapsed disease.^57^–^60^ Interestingly, bortezomib may play a role in the immunomodulation of GVHD: in a preclinical murine model, it down-regulated cytokine synthesis, induced T cell apoptosis, prevented GvHD. Importantly, graft-vs-tumor effects were not affected.^61^,^62^ More recently, Blanco et al showed that bortezomib induced selective depletion of allo-reactive T lymphocytes, decreased the production of Th1 cytokines and allowed the emergence of a suppressor T cell subset.^63^,^64^ Of note, another study has shown that the combination of bortezomib with tacrolimus and methotrexate was very effective in the prevention of GvHD after reduced-intensity allografts from HLA-mismatched unrelated donors.^65^ These findings appear attractive for studies in myeloma patients.

## Conclusions:

Overall, myeloablative allografts have cured a minority of patients who obtained complete clinical remission after transplant. Reduced-intensity and non-myeloablative conditionings represent a clinical and biological breakthrough given that toxicity was greatly reduced and the existence of graft-vs-myeloma effects were indubitably shown. Long-term disease control and disabling chronic GVHD in a subset of patients represent important issues.

If an allograft should be part of first-line treatment plans or of salvage therapy for refractory/relapsed patients is still hotly debated. In newly diagnosed patients with chemosensitive disease, therefore in complete or very good remissions, a non-myeloablative conditioning would safely allow for donor engraftment with a reduced risk of toxicity and would potentially add a curative graft.vs.myeloma effect in a subset of patients. To support this, many reports show that better outcome is associated with chemosensitive disease at transplant and that allografting at an earlier disease phase is associated with stronger graft-vs-myeloma effects.^66^,^67^ This almost unanimously reported observation may be related to an antigen expression profile of potential targets for donor T cells that change through the disease phases. Siegel et al. reported the identification of HLA-A*0201-presented T cell epitopes, derived from the oncofetal antigen-immature laminin receptor protein, in many haematological malignancies.^68^ However, it was interestingly observed that the expression of these antigens on plasma cells was lost over time. Even though very different in design, long-term results of donor-vs-no donor comparisons of the Blood and Marrow Transplant Clinical Trials Network (BMT CTN), the Dutch Hovon, the EBMT, and the German DSMM studies may allow valuable information on the use of up-front allografting.

Other Authors underline the fact that new treatment schemas may likely translate into longer overall survival and would be more inclined to offer an allograft at relapse. In this case, however, disease reduction prior to transplant and a more intense conditioning, rather than a non-myeloablative regimen, would be required despite a higher risk of toxicity.

In conclusion, future studies cannot be designed without the combination of new drugs that may enhance graft-versus-myeloma effects to allow long-term disease control and prolong survival even in patients with high risk disease. Profound cytoreduction before and enhanced graft-versus-myeloma effects after allografts through the immunomodulatory properties of lenalidomide and bortezomib may be key factors to improve clinical outcomes.

Optimal timing of an allograft and dosage of new drugs remain to be determined and should be explored prospectively only in the context of clinical trials and not routinely recommended.

## Figures and Tables

**Table 1.** Myeloablative Conditioning Regimens for Allografting in Multiple Myeloma

**Author**	**Patients**	**Median Age (years)**	**Conditioning**	**Transplant-Related Mortality %**	**Complete Remission %**	**Overall Survival %**
Bensinger et al.	136	43–48 (<60)	Bu, Cy, +Total Body Irradiation	48 (at day 100) 63 (at 1 year)	34	22 (at 5 years)
Barlogie et al.	36	≤55	Melphalan (100 mg/m^2^), Total Body Irradiation (12Gy)	53 (at 1 year)	---	39 (at 7 years)
Reece et al.	26	43	Cy, Total Body Irradiation Bu,Cy Melphalan (100 mg/m^2^), Total Body Irradiation	19 (at day 100)	62	47 (at 3 years)
Alyea et al.	24	46	Cy, Total Body Irradiation (14Gy) Bu,Cy	10	---	55 (at 2 years)
Kulkarni et al.	33	38	Melphalan (110 mg/m^2^), Total Body Irradiation (10.5Gy) Cy, Total Body Irradiation (9.5Gy) Cy, Melphalan Bu,Cy	54	37	36 (at 3 years)
Le Blanc et al.	37	47	Cy, TBI (12Gy) Melphalan (140 mg/m^2^), Total Body Irradiation (10.5Gy) Bu,Cy Others	22	57	32 (at 40 months)
Couban et al.	22	43	Melphalan (160 mg/m^2^), Total Body Irradiation (12Gy) Cy, TBI (12Gy) Bu,Cy	59	50	32 (at 3 years)
Varterasian et al.	24	43	Cy, Total Body Irradiation Melphalan, Total Body Irradiation Bu,Cy, Total Marrow Irradiation Others	25	---	40 (at 3 years)

Abbreviations: Bu: Busulfan; Cy: cyclophosphamide)

**Table 2.** Non-myeloablative/Reduced Intensity Conditioning Regimens for Allografting in Multiple Myeloma

**Author**	**Patients**	**Conditioning**	**Transplant-Related Mortality %**	**Chronic GVHD %**	**Complete Remission %**	**Overall Survival %**
Mohty et al.	41	Bu, Fluda, ATG	17	41	24	62 (at 2 years)
Peggs et al.	20	Total Body Irradiation, Fluda, alemtuzumab	15	---	10	71 (at 2 years)
Einsele et al.	22	Total Body Irradiation (2Gy), Fluda, Cy	23	32	27	26 (at 2 years)
Giralt et al.	22	Fluda, Melphalan (90/140 mg/m^2^)	41	27	32	30 (at 2 years)
Gerull et al.	52	Total Body Irradiation (2Gy), Fluda	17	70	27	41 (at 1.5 years)
Maloney et al.	54	Total Body Irradiation (2Gy)/Total Body Irradiation (2Gy), Fluda	22	60	57	69 (at 5 years)
Lee et al.	45	Melphalan (100 mg/m^2^), Total Body Irradiation (2Gy), Fluda	38	13	64	36 (at 3 years)
Kroger et al.	17	Melphalan (100 mg/m^2^), Fluda, ATG	18	7	73	74 (at 2 years)
Kroger et al.	21	Melphalan (100–140 mg/m^2^), Fluda, ATG	24	12	40	74 (at 2 years)

Abbreviations: Bu: Busulfan; Fluda: fludarabine; ATG anti-thymocyte globulin
